# Inhalation of odors containing DMHF generated by the Maillard reaction affects physiological parameters in rats

**DOI:** 10.1038/s41598-020-70843-z

**Published:** 2020-08-18

**Authors:** Issei Yokoyama, Motoko Ohata, Yusuke Komiya, Jun Nagasao, Keizo Arihara

**Affiliations:** 1 Department of Animal Science, School of Veterinary Medicine, Kitasato University, Towada, 034-8628 Japan; 2 College of Bioresource Sciences, Nihon University, Fujisawa, 252-0880 Japan

**Keywords:** Health care, Nutrition, Chemical biology, Pharmacology, Physiology, Circulation

## Abstract

The effects of odors generated by the Maillard reaction from amino acids and reducing sugars on physiological parameters (blood pressure, heart rate, and oxidative stress levels) in Wistar rats were investigated in the present study. The Maillard reaction samples were obtained from glycine, arginine, or lysine of 1.0 mol/L and glucose of 1.0 mol/L with heat treatment. The odor-active compounds in the Maillard reaction samples were identified using the aroma extract dilution analysis. Among the odor-active compounds identified, 2,5-dimethyl-4-hydroxy-3(2*H*)-furanone (DMHF, FURANEOL and strawberry furanone) had the highest odor activity and its concentration was affected by amino acid types. The Maillard reaction odors generated from glycine or arginine significantly decreased systolic blood pressure and heart rate in rats when inhaled. These physiological effects were associated with DMHF. Furthermore, oxidative stress marker levels in rat plasma were decreased by the inhalation of DMHF. The inhalation of DMHF appears to at least partly affect physiological parameters by decreasing oxidative stress.

## Introduction

The inhalation of odors affects physiological parameters (e.g. blood pressure, heart rate, and blood components). For example, essential oils of lavender and grapefruit change blood pressure via the autonomic nervous system^1,2^. Furthermore, the odor of a mixture containing (*Z*)-3-hexenol and (*E*)-2-hexenal attenuates the cold pressor test-induced blood pressure response in human^3^. Odors may exert their effects through the direct activation of the olfactory system, which sends odor information to the brain, or indirectly through diffusion into the bloodstream after absorption or digestion. The former affects brain functions involved in the autonomic nervous system and immediately induces physiological changes. The latter exerts delayed effects because it requires 20–30 min to diffuse into the bloodstream and reach the brain through the blood–brain barrier. Odors exert their effects on physiological parameters through these pathways via the autonomic nervous system.

The autonomic nervous system alters metabolism by changing physiological parameters, such as blood pressure, heart rate (HR), and oxidative stress^4–7^. Long-term disturbances in physiological parameters also affect many diseases because of increases in oxidative stress^8–10^. Cardiovascular modifications have been associated with an increase in metabolism with oxygen consumption and the promotion of oxidative stress. Hydroperoxides (ROOH), biomarkers of oxidative stress, are produced when reactive oxygen species react with lipids, proteins, and amino acids. Therefore, increases in hydroperoxide levels reflect the extent of oxidative stress in the body^11^. Chronic odor inhalation has been reported to affect oxidative stress levels in the rat brain^12^. Although odors may regulate oxidative stress levels through inhalation, the accumulation of further evidence is needed.

The Maillard reaction, which plays a critical role in flavor and color development in foods subjected to thermal processing^13,14^. The Maillard reaction between amino compounds and reducing sugars very frequently occurs in processed foods such as coffee^15^, beer^16^, bread^17^, soy sauce^18^, and grilled meats^19^. This reaction is of vital importance to the quality of foods because it generates a wide variety of odor compounds that contribute to overall flavor. A number of factors, such as the types of amino acids and sugars, temperature, water activity, time, and pH, markedly affect the rate of formation and the type of Maillard compounds formed.

Although the physiological activities of odors from essential oils have been examined in rats^20^ and human^21^, limited information is currently available on the effects of Maillard reaction odors. We previously reported that the odors generated by the Maillard reaction of meat protein digests with xylose decreased systolic blood pressure (SBP)^22^. Moreover, we identified four major odor-active compounds (2,5-dimethyl-4-hydroxy-3(2*H*)-furanone (DMHF), acetic acid, 2-hydroxy-3-methyl-2-cyclopentenone and 5-methyl-2-pyrazinemathanol) by gas chromatography olfactometry analysis. Since meat protein digests contain various free amino acids, we focused on the amino acid and reducing sugar Maillard reaction system. In the Maillard reaction, all amino acids react under different optimal conditions. Particularly, some amino acids are frequently used for experiments because they advance the reaction. Glycine, which has the simplest structure, or basic amino acids, such as arginine and lysine, were shown to sufficiently advance the Maillard reaction^23–25^. Amino acids have been commercially utilized as food additives during the processing and cooking of various foods to preserve flavor or enhance taste, appearance, or other qualities.

The aim of the present study was to investigate the effects of odors generated by the Maillard reaction from amino acids and glucose on physiological parameters in a rat model. The odor-active components in the Maillard reaction samples were identified by the aroma extract dilution analysis (AEDA) method. In addition, the effects of the inhalation of these odor components on SBP, HR and oxidative stress were examined.

## Results

### Identification and quantification of odor-active compounds in ariginine/lysine-glucose Maillard reaction samples by AEDA

The potent odor-active compounds of each Maillard reaction sample were determined by AEDA technique and the concentration of each odor compound is shown in Table 1. We previously identified ten odor-active compounds in glycine-glucose Maillard reaction sample and the highest flavor dilution (FD) factors were 2,3-dimethylpyrazine and DMHF^26^. In the present study, five odor-active compounds were detected from arginine-glucose system, and methylpyrazine, 2,3-dimethylpyrazine, acetic acid, DMHF, and 2,3-dihydro-5-hydroxy-6-methyl-4*H*-pyran-4-one were tentatively identified based on mass spectra. DMHF was the odor compound showing the highest FD factor in arginine-glucose Maillard reaction sample (FD factor = 4,096). Lysine-glucose Maillard reaction sample contained seven odor-active compounds, and the FD factor of DMHF was the highest (FD factor = 65,536). DMHF showed the highest FD factor in all Maillard reaction samples. However, its concentration varied according to the types of amino acids (glycine: 57.7 ppm, arginine: 67.5 ppm, lysine: 228.4 ppm). In addition, the odor activity value (OAV) of the identified odor compounds was calculated. In the OAV obtained from previous study^26^, 2,3,5-trimethylpyrazine was the highest (OAV = 8,000), followed by DMHF (OAV = 1923). On the other hands, DMHF had the highest values (arginine = 2,250, lysine = 7,613), followed by pyrazines and acetic acid in the arginine/lysine-glucose Maillard reaction samples.

**Table 1:** FD factors and concentrations of odor-active compounds in Maillard reaction samples.

KI^a^	RT^b^	Odor compound	Odor quality^c^	Odor threshold^d^ (ppm)	FD factor			Concentration^e^ (ppm)			Odor activity value (OAV)^f^		
					Gly^k^	Arg	Lys	Gly^k^	Arg	Lys	Gly	Arg	Lys
1269	22.3	Methylpyrazine	Nutty, roasted	0.06	–^g^	1	–^g^	–^g^	36.9	–^g^	–^g^	615	–^g^
1347	27	2,3-Dimethylpyrazine	Nutty, savory	0.02	1,024	64	64	0.92	0.83	1.48	46	41.5	74
1406	30.2	2,3,5-Trimethylpyrazine	Peanut, green	0.001	16	–^g^	16	8	–^g^	1.25	8,000	–^g^	1,250
1454	31.9	Acetic acid	Sour	1	1	1	1	7.42	49	44	7.42	49	44
1597	36.4	Unknown^h^	Popcorn	–^i^	–^g^	–^g^	16	Trace	Trace	Trace	–^j^	–^j^	–^j^
1888	47.3	2-Hydroxy-3-methyl-2-cyclopenten-1-one	Sweet, burnt	–^i^	–^g^	–^g^	256	–^g^	–^g^	1.09	–^j^	–^j^	–^j^
2039	54.5	2,5-Dimethyl-4-hydroxy-3(2*H*)-furanone	Caramel, sweet	0.03	4,096	4,096	65,536	57.7	67.5	228.4	1,923	2,250	7,613
2258	60.4	2,3-Dihydro-5-hydroxy-6-methyl-4*H*-pyran-4-one	Sweet	–^i^	4	4	256	–^g^	–^g^	–^g^	–^j^	–^j^	–^j^

*Gly* glycine-glucose, *Arg*: arginine-glucose, *Lys* lysine-glucose.

^a^Kovats’ index on the DB-Wax capillary. ^b^Retention time. ^c^Odor quality assigned during AEDA. ^d^Odor threshold in water. Information from the National Institute of Health Science (Japan). ^e^Concentration of the odor compound in the Maillard reaction sample. ^f^The ratio between the concentration of individual odor compounds in the sample and the threshold. ^g^The odor compound was too weak to be detected by GC–O. ^h^The compound was not purchased or quantified. ^i^Data not available. ^j^The OAV was not determined. ^k^The reference data were adapted from Zhou et al. (2016).

### Effects of odors from Maillard reaction samples on SBP and HR

**Figure 1:**
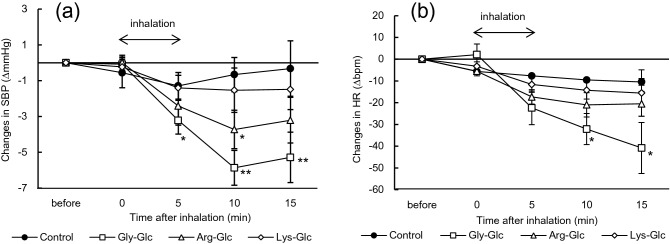
Effects of odors from each model Maillard reaction sample on SBP and HR. (**a**) Changes in SBP (mean ± SEM, n = 5 per each group); (**b**) changes in HR (mean ± SEM, n = 5 per each group) in Wistar rats after the inhalation of each Maillard reaction sample. SBP and HR in rats were measured during the 15-min trial. Odor inhalation was performed before the circadian rhythm dark period and repeated three times (once a week for each sample). Rats were able to access water and chow diets ad libitum unless SBP and HR were measured. Differences in SBP and heart rate were statistically analyzed by a two-way ANOVA with Dunnett’s test. *SBP* systolic blood pressure, *HR* heart rate, *Arg* arginine, *Lys* lysine, *Gly* glycine, *Glc* glucose; *p < 0.05, **p < 0.01 (vs. control).

SBP and HR decreased after the inhalation of volatiles in the headspace of glycine/arginine-glucose Maillard reaction samples, but lysine-glucose did not significantly change SBP or HR (Fig. 1a). Regarding the glycine-glucose Maillard reaction samples, SBP significantly decreased 5 min after inhalation (*p* < 0.05), and low SBP was maintained during the 15-min trial (*p* < 0.01). A significant difference was observed in SBP 10 min after the inhalation of arginine-glucose Maillard reaction sample (*p* < 0.05). HR also decreased after the inhalation of Maillard reaction samples, and significant differences were noted in HR 10 and 15 min after the inhalation of the glycine-glucose Maillard reaction sample (Fig. 1b).

### Effects of different DMHF concentrations on SBP and HR

**Figure 2:**
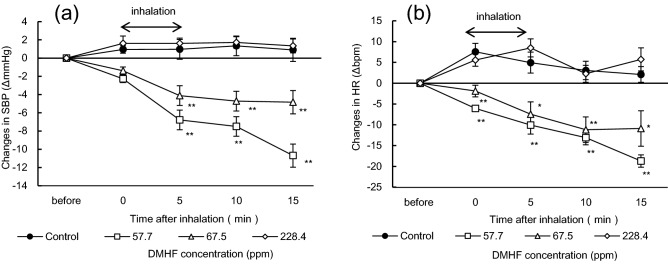
Effects of DMHF inhalation on SBP and HR. (**a**) Changes in SBP (mean ± SEM, n = 5 per each group); (**b**) changes in HR (mean ± SEM, n = 5 per each group) in Wistar rats after the inhalation of different concentrations of DMHF. Each DMHF solution was dissolved in distilled water. SBP and HR in rats were measured during the 15-min trial. Odor inhalation was performed before the circadian rhythm dark period and repeated three times (once a week for each sample). Rats were able to access water and chow diets ad libitum unless SBP and HR were measured. Differences in SBP and heart rate were statistically analyzed by a two-way ANOVA with Dunnett’s test. *SBP* systolic blood pressure, *HR* heart rate; *p < 0.05, **p < 0.01 (vs. control).

DMHF solutions adjusted to 57.7 and 67.5 ppm decreased SBP and HR through inhalation (Fig. 2a). A significant change in SBP was observed from 5 min (*p* < 0.01) after inhalation until the end of the trial. However, SBP was not changed at 228.4 ppm DMHF during the trial. HR was also significantly decreased by the inhalation of DMHF (57.7 and 67.5 ppm), while DMHF at 228.4 ppm did not cause any significant changes (Fig. 2b).

### Changes in SBP and HR after the inhalation of other odor-active compounds

**Figure 3:**
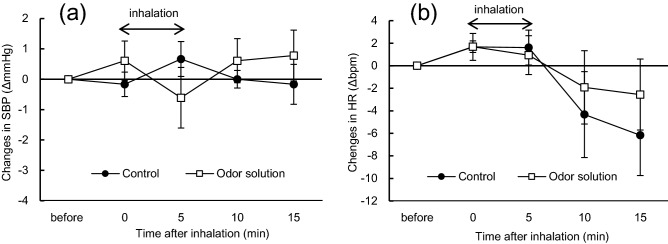
Changes in SBP and HR after the inhalation of distilled water as the control or constructed odor solution without DMHF. (**a**) Changes in SBP (mean ± SEM, n = 5 per each group); (**b**) changes in HR (mean ± SEM, n = 5 per each group) in Wistar rats. Odor solution was constructed with acetic acid (7.42 ppm), 2,3-dimethylpyrazine (0.92 ppm) and 2,3,5-trimethylpyrazine (8.00 ppm). These odor compounds concentration were referred from Zhou et al. (2016) and dissolved in distilled water. SBP and HR in rats were measured during the 15-min trial. Odor inhalation was performed before the circadian rhythm dark period and repeated three times (once a week for each sample). Rats were able to access water and chow diets ad libitum unless SBP and HR were measured. Differences in SBP changes between the control and odor groups were not significantly different by a two-way ANOVA. *SBP* systolic blood pressure, *HR* heart rate.

To confirm that physiological activities were changed by DMHF, rats inhaled other individual odor-active volatiles identified in glycine-glucose sample. Three odor-active compounds in glycine-glucose Maillard reaction sample (acetic acid: 7.42 ppm, 2,3-dimethylpyrazine: 0.92 ppm, 2,3,5-trimethylpyrazine: 8.00 ppm) were mixed. The effects of the mixture on SBP and HR were then investigated and the results obtained are shown in Fig. 3. SBP and HR did not significantly differ from the control during the 15-min trial.

### Effects of DMHF inhalation on oxidative stress levels in rat plasma

**Figure 4:**
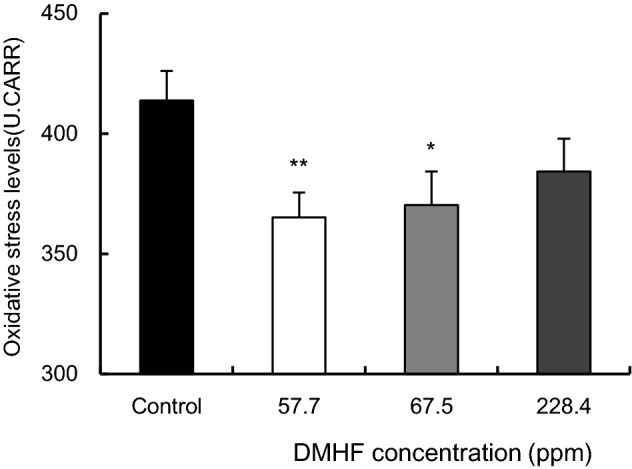
Effects of DMHF inhalation on oxidative stress levels in rat plasma. After inhalation of each DMHF solution, rats were sacrificed after overnight fasting, and blood samples were collected for d-ROMs test. Plasma was prepared by centrifugation at 4 °C and 3,000 rpm for 10 min. The results of the d-ROMs test are expressed as Carratelli Units (CARR U), where 1 U CARR corresponds to 0.08 mg/100 mL H_2_O_2_. Data are means ± SEM (n = 4 or 5 per each group). Differences in hydroperoxide levels between the control and DMHF solution were statistically analyzed by Dunnett’s test. *p < 0.05, **p < 0.01 (vs. control).

To investigate whether the inhalation of DMHF affects oxidative stress, hydroperoxide levels in rat plasma were measured using the d-ROMs test. Figure 4 shows the results of the d-ROMs test after the DMHF inhalation trial. Hydroperoxide levels in rat plasma were significantly lower after the inhalation of DMHF (57.7 and 67.5 ppm) than in the control group (*p* < 0.01 or 0.05). Although the inhalation of DMHF at 228.4 ppm decreased hydroperoxide levels, a significant change did not observed.

## Discussion

The aim of the present study was to investigate the effects of odors generated by the Maillard reaction from amino acids and glucose on physiological parameters through inhalation. The odor compositions of two Maillard reaction samples (arginine/lysine-glucose) were analyzed to identify the odor compounds and evaluate the odor activity. As shown in Table 1, the AEDA technique revealed that DMHF, pyrazines and 2,3-dihydro-5-hydroxy-6-methyl-4*H*-pyran-4-one showed the high FD factor in the identified odor compounds. Previous studies reported that the degree of the Maillard reaction differed based on the types of amino acids^27,28^. The ε-amino groups of lysine residues or amino acid side chains, such as arginine, would be highly reactive^29^. The concentration of DMHF, the highest FD factor in the all Maillard reaction samples, varied according to amino acids (glycine: 57.7 ppm, arginine: 67.5 ppm, lysine: 228.4 ppm). Furthermore, OAV of DMHF was also high in the Maillard reaction samples. OAV provide another approach to identify odor compounds which contribute to overall odor of complex mixtures. It is calculated as the ratio between the concentration of individual odor compounds in the sample and the threshold. The FD factor and OAV of DMHF was high compared with other odor compounds, which suggests that DMHF is one of the major odor-active compounds in the Maillard reaction sample.

We have paid particular attention to physiological activities of DMHF through inhalation^22,26,30–33^. DMHF is present in natural fruits (e.g., strawberries and pineapple)^34,35^ and processed foods^36–38^. It is also known as FURANEOL or strawberry furanone in the flavor industry. In the Maillard reaction, the main precursors for DMHF are aldehydes derived from reducing sugars and methylglyoxal^39^. Also, DMHF is generated by the caramelization reaction^40^. However, the temperature at which the caramelization reaction occurs according to the sugar type^41^. The caramelization reaction of glucose is caused at approximately 160 °C. It was suggested that the generation of DMHF under heat treatment at 90 °C did not mainly derive from the caramelization reaction in this study.

In a subsequent rat model experiment, odors from glycine/arginine-glucose Maillard reaction sample decreased SBP and HR through inhalation. These physiological changes were observed from 5 min after odor inhalation. The autonomic nervous system plays a crucial role in the regulation of SBP and HR. The activation of sympathetic nerve activity increases SBP and HR^42,43^. Odors from cats, a well-known predator of rats, were previously shown to increase blood pressure 5–10 min after their inhalation in rats^44^. On the other hand, these parameters were decreased by parasympathetic nerve activity^20^. The present results suggest that odors affect the autonomic nervous system and then induce physiological parameters. Also, DMHF with high FD factor and OAV would be a key responsible agent for the physiological activities. We previously found that the inhalation of DMHF at 5.7 ppm to Wistar rats decreased SBP^32^ and promoted appetite^45^ via parasympathetic nerve activity. As shown in Fig. 3, SBP and HR decreased after the inhalation of DMHF as well as glycine/arginine-glucose Maillard reaction samples. Furthermore, odors composed of the odor-active compounds (2,3-dimethylpyrazine, trimethylpyrazine, and acetic acid) in the glycine-glucose Maillard reaction sample did not affect SBP and HR. These results suggest that DMHF is one of the causative agents affecting physiological changes. However, the synergistic effects were not investigated in this study. It is known that the odors are caused by interaction of the odor components. Hwang et al.^46^ reported that pyrazines, pyridines and pyrroles are found in the lysine-glucose system at high pH condition. There is a possibility that other chemical compounds not identified in the lysine and glucose Maillard reaction sample may affect physiological parameters. Therefore, it is necessary to investigate the physiological evaluation with other odor-active compounds.

On the other hand, DMHF was also detected in lysine-glucose Maillard reaction sample; however SBP and HR did not change following its inhalation. Similarly, the inhalation of DMHF at 228.4 ppm did not significantly change physiological parameters. Odor concentrations markedly affect physiological responses. Odors perceived as pleasant generally induce positive effects, whereas unpleasant ones may have the opposite effects^47^. (*Z*)-5-tetradecen-1-ol evoked attraction behavior in mice at low concentrations, but aversion at higher concentrations^48^. In humans, the inhalation of DMHF solution at a range between 2.5 to 58 ppm increased alpha brainwaves, which are associated with relaxation. However, beta brainwaves, which are associated with being busy and anxious or more active and fresher moods, increased at a concentration of 100 ppm^26^.

After the inhalation of DMHF, rat plasma was collected to assess hydroperoxide levels using the d-ROMs test. The inhalation of DMHF (57.7 and 67.5 ppm) decreased hydroperoxide levels in rat plasma. The activation of sympathetic nerve activity has been shown to increase oxidative stress levels^7^. Moreover, oxidative stress levels were found to be elevated in rats exposed to cat odors^12^. Accordingly, the sympathetic nervous system increases oxidative stress and its activity is influenced by odors. Vassalle et al.^49^ reported that hydroperoxide levels are an indicator of cardiovascular diseases caused by the long-term activation of sympathetic nerve activity. Thus, changes in autonomic nerve activities indirectly affect hydroperoxide levels, suggesting that the inhalation of DMHF decreased hydroperoxide levels through the suppression of sympathetic nerve activity. On the other hand, DMHF exhibited antioxidative activity following its oral administration to mice^50,51^. Usually, such effect increases with the amount of ingestion. The inhalation of DMHF at 228.4 ppm decreased hydroperoxide levels, but a significant change was not observed. DMHF seemed to affect hydroperoxide levels through the changes in autonomic nerve activity rather than its antioxidative effect.

DMHF also stimulates feeding behavior via the autonomic nervous system^45^. The oral ingestion is a factor for the change of physiological outcomes and oxidative stress. It has been reported that constant hyperglycemia after the ingestion leads to increase in oxidative stress^52^. However, hydroperoxide levels did not increase after DMHF inhalation, suggesting that the influence of oral ingestion is relatively small in this study. Further investigations are required to examine changes in antioxidative activity through inhalation (e.g., DMHF levels in blood and long-term inhalation) and the relationship between rat behavior and physiological responses according to the DMHF concentration. To date, few studies have investigated the effects of odors on oxidative stress through inhalation, and the findings of future studies will provide further evidence to support odor inhalation regulating oxidative stress. The present results suggest that DMHF in Maillard reaction samples affected physiological parameters at least partly by decreasing oxidative stress.

In the present study, DMHF concentrations were affected by the types of amino acids and DMHF was detected at a high concentration using lysine. A smaller amount of lysine may control DMHF concentrations. In food processing and cooking, amino acids are utilized to increase the nutritive value and palatability of foods^53^. In addition, DMHF generated by the Maillard reaction affects physiological parameters and its concentration is controlled by amino acids. During food processing and cooking, the addition of amino acids considering physiological outcomes (e.g., taste perception) may be applied to the functional properties of foods. In conclusion, Maillard reaction odors generated from amino acids and reducing sugars (glycine/arginine-glucose) decreased SBP and HR in rats following their inhalation. Among the potent odor compounds examined, DMHF was identified as a potential odor-active compound affecting physiological parameters and decreasing hydroperoxide levels in rat plasma following its inhalation. Amino acids, such as glycine, arginine, and lysine, affect DMHF concentrations and are available for controlling DMHF during the processing and cooking of foods.

## Materials and methods

### Chemicals and reagents

Acetic acid (purity: > 99%), arginine, 2-hydroxy-3-methyl-2-cyclopentenone (purity: > 98%), diethyl ether, d-glucose, lysine, glycine, pentane, methylpyrazine (purity: > 99%), and sodium carbonate were purchased from Kanto Chemical Co. (Tokyo, Japan). 2,3-Dimethylpyrazine (purity: > 95%), DMHF (purity: > 98%), methyldecanoate (purity: > 98%), and 2,3,5-trimetylpyrazine (purity: > 98%) were purchased from Tokyo Chemical Industry Co. (Tokyo, Japan).

### Animals

The animals were housed as described previously^45^. Twenty 8-week-old male Wistar rats were purchased from CLEA Japan Inc. (Tokyo, Japan). Rats were housed in plastic cages in an animal room at 23 ± 2 °C and 50 ± 10% humidity under an artificial lightning system of 12-h light and 12-h darkness (lights on from 07:00 to 19:00). Rats were able to access water and chow diets ad libitum during the experimental period. All animal experiments were conducted in strict accordance with the recommendations in the Guidelines for the Proper Conduct of Animal Experiments published by the Science Council of Japan, and with the approval of the Animal Care and Use Committee of Kitasato University (approval no. 16-169).

### Preparation of Maillard reaction samples

The Maillard reaction samples were prepared as described previously^26^. Equimolar solutions of glucose (1.0 mol/L) and arginine, glycine, or lysine (1.0 mol/L) were prepared in 0.25% (w/v) sodium carbonate buffer and mixed. Sodium carbonate buffer was used because of its common use in food processing. pH was adjusted to 9 using NaOH or HCl. Each mixture was poured into screw-capped glass tubes and was heated at 90 °C for 30 min in an oil bath. After the heat treatment, three mixtures were immediately cooled on ice and stored at 4 °C until further experiments.

### Quantification of potent odor compounds in Maillard reaction samples by a gas chromatography (GC) analysis

The potent odor compounds in Maillard reaction samples were quantified as described previously^26^. Twenty-five milliliters of Maillard reaction samples (glycine, arginine, and lysine) were diluted with distilled water (25 mL) and methyl decanoate (final concentration: 0.5 ppm) was added as an internal standard (IS). Each solution was passed through a glass column packed with 5 g of a porous polymer resin (Tenax TA, GL Sciences Inc., Tokyo, Japan) and volatile components were adsorbed to the resin. Volatile components were eluted with 100 mL diethyl ether/pentane (1:1) and purified by solvent-assisted flavor evaporation (SAFE, Kiriyama Laboratory, Tokyo, Japan) at 50 °C under a vacuum (10^−6^ Pa). The volatile fraction was dried over anhydrous sodium sulfate and concentrated in a water bath (40 °C) for GC and a GC–mass spectrometry (GC–MS) analysis. A Shimadzu GC2010 Plus GC equipped with a flame-ionization detector (FID) and fused silica capillary column (DB-Wax, 60 m × 0.25 mm i.d., J&W, Agilent Technologies, Wilmington, USA) was used for the GC analysis, while the GC–MS analysis was performed with a Shimadzu QP-2010 mass spectrometer combined with a Shimadzu GC-2010 gas chromatograph fitted with the same column. The temperature of the oven was maintained at 40 °C for the first 5 min, then programmed to reach 220 °C at a rate of 3 °C min^−1^, and maintained at 220 °C for 20 min. The flow rate of the carrier gas (helium) was 1.5 mL min^−1^ and the injection and detection temperatures were 220 °C. MS was performed in the electron impact mode with an ionization voltage of 70 eV and ion source temperature of 200 °C. Compounds were identified by their agreement with Kovats’ GC retention indices and their mass spectra with authentic standards. The IS ratio for each compound was assessed by the ratio of the corresponding GC peak area versus the peak area of the IS. Calibration standards were prepared by adding increasing quantities of acetic acid (5–20 ppm), 2-hydroxy-3-methyl-2-cyclopentenone (10–500 ppm), 2,3-dimethylpyrazine (10–500 ppm), DMHF (10–500 ppm), methylpyrazine (10–500 ppm), and 2,3,5-trimethylpyrazine (10–500 ppm) to 100 mL of distilled water.

### Identification of potent odor compounds in Maillard reaction samples by AEDA

Potent odor compounds in Maillard reaction samples were assessed by AEDA. AEDA was carried out with the method our previous report^26^. Each sample (125 mL) was diluted with distilled water (125 mL) and the volatile concentrate from each solution was obtained using the procedure described above. The concentrate obtained was analyzed by GC–olfactometry (GC–O) and GC–MS. In the GC–O system, the GC eluate of the odor concentrate was split to a FID and sniffing port. The experimental conditions of GC and GC–MS are described above. After stepwise four-fold dilutions of the concentrate with diethyl ether/pentane (1:1), GC–O was performed for each diluted sample. The AEDA analysis was performed by three panelists. Significant odorous peaks in the eluate were identified by evaluating FD factors.

### Inhalation of odors from Maillard reaction solutions for SBP and HR measurements

SBP and HR were measured by the tail cuff method with a non-invasive and programmed electronic sphygmomanometer (BP-98, Softron Co., Tokyo, Japan). A 5-mL aliquot of distilled water (control) or each Maillard reaction odor sample was deposited into a two-neck flask; air was pumped into one neck and the vapor phase from the flask was ejected from the other. After inhalation of the vapor for 5 min, rats were left to stand for 10 min. SBP and HR in rats were measured during the 15-min trial. Odor inhalation was performed before the circadian rhythm dark period and repeated three times (once a week for each sample). Rats were premeasured blood pressure and then removed abnormal rats.

### Inhalation of odor compounds in Maillard reaction samples

Three DMHF solutions (57.7, 67.5, 228.4 ppm) were inhaled by 8-week-old Wistar rats. After inhalation three times, rats were sacrificed after overnight fasting, and blood samples were collected for further experiments. Plasma was prepared by centrifugation at 4 °C and 3,000 rpm for 10 min and then stored at − 80 °C until used.

To support the effect of DMHF, other three odor-active compounds in the glycine and glucose Maillard reaction sample were dissolved in distilled water (acetic acid: 7.42 ppm, 2,3-dimethylpyrazine: 0.92 ppm, and 2,3,5-trimethylpyrazine: 8.00 ppm). These odor concentration were adopted our previous study^26^, and odor solution was inhaled by Wistar rats. SBP and HR were measured by the method described above.

### Evaluation of oxidative stress levels in rat plasma

Oxidative stress levels were evaluated by the d-ROMs test (Wismerll, Co., Ltd., Tokyo, Japan). The experimental procedure for d-ROMs test was referred to the previous study^54^. Rat plasma was diluted in an acidic buffered solution (pH 4.8). A compound (chromogen) that has the ability to change its color when it is oxidized by hydroperoxyl and alkoxyl radicals was then added to this solution. The chromogenic substrate used in the d-ROMs test was *N*,*N*,-diethyl paraphenylenediamine, the quantity of which was assessed by means of the photometer. The results of the d-ROMs test are expressed as Carratelli Units (CARR U), where 1 U CARR corresponds to 0.08 mg/100 mL H_2_O_2_.

### Statistical analysis

Results are expressed as the mean ± standard error of mean (SEM). A two-way analysis of variance (two-way ANOVA) was performed to assess whether SBP and HR were significantly affected by the inhalation of odor components. Comparisons of SBP, HR, and oxidative stress levels between control and odor groups were performed using Dunnett’s test.
